# Evaluation of Biomarkers of NAFLD in a Cohort of Morbidly Obese Patients

**DOI:** 10.1155/2011/369168

**Published:** 2011-06-23

**Authors:** Julia Kälsch, Lars P. Bechmann, Hagen Kälsch, Martin Schlattjan, Jochen Erhard, Guido Gerken, Ali Canbay

**Affiliations:** ^1^Division of Gastroenterology and Hepatology, University Hospital Essen, 45122 Essen, Germany; ^2^Division of Cardiology, University Hospital Essen, 45122 Essen, Germany; ^3^Department of Surgery, Evangelisches Krankenhaus Dinslaken, 46535 Dinslaken, Germany

## Abstract

Hepatocyte apoptosis is a key event in nonalcoholic fatty liver disease (NAFLD), and serum apoptotic markers are emerging as surrogate markers for NAFLD. We studied the role of caspase-cleaved cytokeratin18 in the diagnosis of fibrosis in a cohort of 127 morbidly obese patients and also performed a review of the literature biomarkers of NAFLD and fibrosis. Here, we found that cleaved caspase 18 correlated with liver steatosis and liver injury as assessed by serum transaminase levels. Furthermore, hepatocyte apoptosis as assessed by cleaved CK18 and TUNEL staining correlated with the extent of fibrosis as assessed by Sirius Red staining and serum hyaluronic acid. These results underscore the important role of hepatocyte apoptosis in the pathogenesis of fibrosis in NAFLD, which led to the utilization of surrogate markers for apoptosis in the noninvasive diagnosis of NAFLD. We furthermore reviewed current literature of biomarkers of NAFLD and fibrosis.

## 1. Introduction

Nonalcoholic fatty liver disease (NAFLD) is the most common chronic liver disease in Europe and the US with a prevalence of up to 30% [1]. It is associated with obesity and diabetes and is thus considered to be the hepatic manifestation of the metabolic syndrome [2]. NAFLD ranges from simple hepatic steatosis, characterized by triglyceride accumulation in hepatocytes, to Nonalcoholic steatohepatitis (NASH), which may be associated with fibrosis and progression to cirrhosis or hepatocellular carcinoma [3]. While the diagnosis of NAFLD is based on histology and patients history, the increase in prevalence demands noninvasive methods for diagnosis and surveillance. 

Hepatocyte apoptosis was identified as a key feature of NAFLD and correlates with disease severity [4]. Cytokeratin 18 (CK18) is an intermediate filament expressed in single-layer epithelial tissues. During apoptosis, caspase-cleaved CK18 is released into the cytoplasm and released into the serum after cell death. Therefore, soluble forms of extracellular CK18 in the serum are utilized to quantify activity of cell death. Wieckowska et al. quantified cleaved CK18 fragments in patients with NAFLD and found a correlation with the occurrence of liver fibrosis and hepatic inflammation. [5, 6] The published results of a multicenter validation study by Feldstein et al. revealed CK18 fragments as a predictor of NASH versus simple steatosis [7].

Hyaluronic acid is a glycosaminoglycan, distributed throughout epithelial tissues. Most of its disassembly takes place in endothelial liver cells. Because of the lack of function, fibrosis and cirrhosis lead to impaired clearance of hyaluronic fragments. Several studies could show a correlation between serum hyaluronic acid and fibrosis stage in chronic liver diseases, including NAFLD [8, 9].

Here, we evaluated caspase-cleaved CK18 and hyaluronic acid as biomarkers for NAFLD and fibrosis in a cohort of 127 patients who underwent bariatric surgery and compared these results to the histological diagnosis, quantified by NAFLD-activity score (NAS), as established by Kleiner et al. [10]. In line with recent publications, we observed a clear correlation between caspase-cleaved CK18 and liver steatosis. It was noticed that high hepatocellular apoptosis rates correlate with fibrosis in NASH patients. Further current literature review was also conducted on noninvasive markers of NAFLD and fibrosis.

## 2. Materials and Methods

### 2.1. Patients

A total of 127 morbidly obese patients (mean age: 45 ± 10; 78.7% females, mean BMI: 52 ± 8) who underwent bariatric surgery at a center for bariatric surgery were included. Indication for bariatric surgery was made according to National Institutes of Health (NIH) guidelines (BMI ≥ 40 kg/m^2^  or ≥ 35 kg/m^2^, plus comorbidities). Subjects reporting excess alcohol consumption (>20 g/day in males or >10 g/day in females) indicating alcoholic liver disease were excluded. The surgeon's choice—that is, adjustable gastric band, Roux-Y, or gastric bypass surgery—was based on the current guidelines as adapted to the patient's clinical conditions and comorbidities as well as on clinical experience. Wedge liver biopsies were taken during the procedure. A control group of 10 healthy volunteers (7 males; 3 females/median age: 26 ± 7.6 years) had an average BMI of 22.4 ± 0.82 kg/m. This study was approved by the ethics committee (Institutional Review Board) of the University Hospital Essen. Patients volunteering were informed about intraoperative risks and benefits of wedge liver biopsy and provided informed consent.

### 2.2. Sample Preparation

The degree of NAFLD was quantified according to the NASH Scoring System. NAS score of ≥5 was defined as NASH. CK18 serum levels and hyaluronic acid were assessed in the sera of patients and healthy controls using the M30-Apoptosense (Peviva, Bromma, Sweden) and hyaluronic acid (Wako Chemicals, Neuss, Germany) ELISA kits. The M30-Apoptosense assay detects a specific epitope of caspase-cleaved CK18, which will further be referred to as M30. All procedures were conducted according to the manufacturers' instructions.

### 2.3. Statistics

All data shown are mean ± SEM, if not stated otherwise. Patients with and without NASH were compared regarding metabolic characteristics, transaminases, NAS, M30, and hyaluronic acid levels using independent samples *t*-tests (Mann-Whitney test). Medline research was performed in January 2011 with the search terms “serum markers NAFLD” [11]. Analyses were performed with with SPSS 15.0.1, Version 2006 (SPSS, Chicago, IL, USA) and Graph Pad, version 5.03 (Graph Pad, Graph Pad Software Inc., CA, USA).

## 3. Results

### 3.1. Patient Characteristics

The clinical characteristics of the 56 patients with NASH (NAS ≥ 5) in comparison to 71 NAFL patients (NAS < 5) are stated in Table 1. In patients with NASH, risk factors of the metabolic syndrome as triglycerides and fasting glucose were higher in comparison to patients with NAFL. However, there were no significant differences in between the two groups.

### 3.2. Caspase-Cleaved CK18 Predicts Steatosis and Hepatocellular Injury

**Figure 1:**
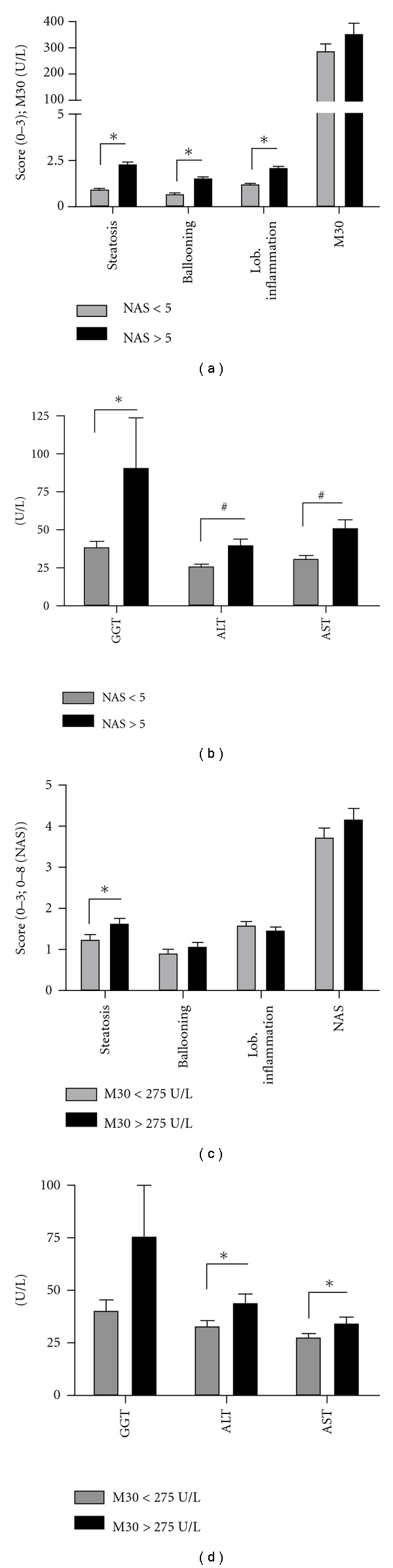
NAS versus M30 assay. As expected, the NAS score correlates with its individual parameters steatosis, ballooning, and lobular inflammation. M30 is higher in patients with histologically established NASH (NAS ≧ 5;) (a). Serum transaminase levels are higher in patients with NASH (b). M30 correlates with histological steatosis, but not lobular inflammation (c). ALT and AST are significantly higher in patients with M30 ≧ 275 U/L (d). (*= *P* < .05; ^#^= *P* < .01).

**Figure 2:**
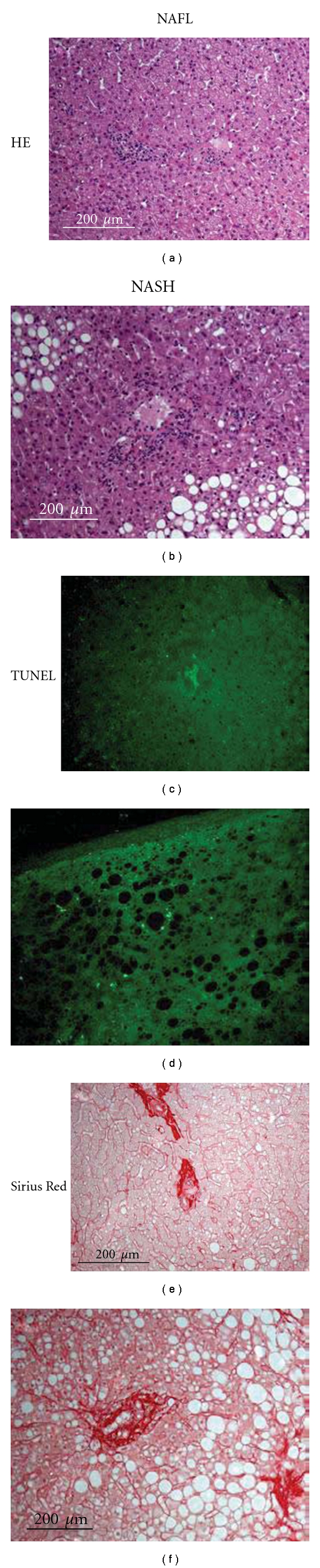
Histological assessment in NAFLD. NASH is histologically defined by increased steatosis, fibrosis, and lobular inflammation (a/b). Apoptosis is a key feature of NASH, which is accompanied by an increase in TUNEL positive cells (c/d). Hepatocyte apoptosis triggers HSC activation and thus fibrogenesis. Representative slides with quantification of collagen by Sirius red staining (e/f).

As the NAS score consists of the individual scoring values for steatosis, ballooning, and lobular inflammation, a NAS ≥ 5 was accompanied with significantly higher scores for the individual characteristics of NAFLD. As expected, M30 was elevated in NASH patients (Figures 1(a), 2(a), and 2(b)). Accordingly, serum transaminase levels were elevated in NASH patients (Figure 1(b)). The artificial cut-off for M30 to predict NASH (275 U/L), established and validated by the Feldstein group, in our cohort corresponded with histological steatosis, but not lobular inflammation [7]. In serological NASH, ballooning and NAS score were only modestly elevated (Figure 1(c)). However, alanine aminotransferase (ALT) and aspartate aminotransferase (AST) levels were significantly higher in patients with plasma levels of M30 ≥275 U/L, indicating hepatocellular damage, despite no histological signs of lobular inflammation (Figure 1(d)).

### 3.3. Fibrosis in NAFLD Correlates with Apoptosis, Not with Histological Features of NASH

**Figure 3:**
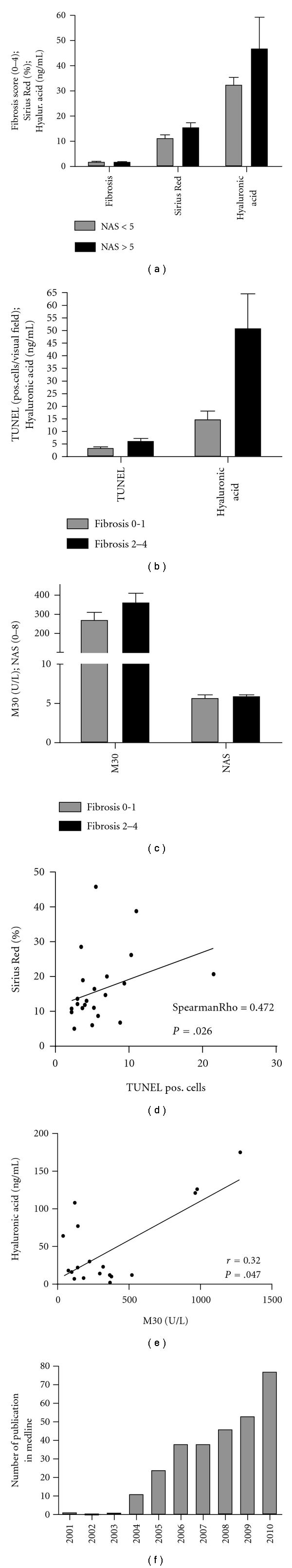
Association between apoptosis and fibrosis. Although fibrosis, as quantified by a pathologist in H&E sections is not increased in NASH, collagen staining and serum hyaluronic acid indicate more fibrosis in NASH (a). Hepatocyte apoptosis and serum hyaluronic acid are increased in advanced fibrosis (b, c). Cell death rate is associated with fibrotic areal in histological slides from NASH patients (d). Hyaluronic acid is correlated with serum cleaved CK18 (e). Linear increase in publications/year on serum markers of NAFLD (f).

Interestingly, about two-third of our cohort had stage-2 fibrosis (Figures 2(a) and 2(b)). We did not find an increase in fibrosis score in patients with a NAS ≥ 5. However, morphometrically quantified liver collagen (Sirius Red) and serum hyaluronic acid were both elevated in histological NASH versus NAFL (Figures 3(a), 2(e), and 2(f)). Advanced fibrosis was further associated with more TUNEL positive cells per visual field in liver sections, and serum hyaluronic acid was dramatically increased in patients with advanced fibrosis (Figures 3(b), 2(c), and 2(d)). Accordingly, M30 levels were higher in patients with advanced fibrosis, while the NAS score was not different between the groups at all (Figure 3(c)). This supports the above-mentioned observation that fibrosis is associated with hepatocellular apoptosis, rather than lobular inflammation. In this context, the number of TUNEL positive cells in livers was significantly correlated with the percentage of Sirius Red positive area in the visual field and M30 was significantly correlated with serum hyaluronic acid (Figure 3(e)). 

## 4. Discussion and Review

In our cohort of 127 morbidly obese patients, caspase-cleaved CK18 correlated with hepatic steatosis but not lobular inflammation as assessed by histological scoring. Still, AST and ALT were significantly higher in patients with CK18 levels above the established cut-off for NASH, indicating a discrepancy between histological assessed inflammation and actual liver injury. Interestingly, the degree of fibrosis did not correspond to the NAS score, while a high apoptosis rate, as assessed by TUNEL staining in liver tissue and assessment of serum caspase-cleaved CK18, correlated with increased fibrosis. This is in line with recent publications, that hepatocyte apoptosis, rather than hepatic inflammation, is a key trigger of hepatic stellate cell activation and thus fibrogenesis [12, 13]. Taken together, our data indicate that biomarkers in NAFLD might reveal more information than standard histological scoring. In the following, we will review the emerging data on serum markers in NAFLD, a field that is continuously growing (Figure 3(f)). 

To date, liver biopsy and histological assessment of liver steatosis, ballooning, and lobular inflammation in H&E staining remain the gold standard for diagnosis of NASH [10]. However, despite a low rate of overall complications, percutaneous liver biopsy remains an invasive procedure with the risk of potentially lethal hemorrhage and infections and is further complicated in the growing number of obese patients with NAFLD [14, 15]. Furthermore, several studies suggested a relatively high rate of sample and interpretational errors [16]. Given the increasing prevalence of NAFLD, noninvasive markers for NAFLD are a promising approach not only for screening reasons.

In 2006, Wickowska et al. quantified soluble caspase-cleaved CK18 with a specific M30-ELISA and found a correlation with the histological staging in NAFLD patients [5]. This was validated by the same group and in a cohort of patients who underwent bariatric surgery, an M30-cutoff value of 275 U/L was established to discriminate simple steatosis from NASH [7, 17]. We and others were able to show that M30 correlates with fatty acid transporter expression in liver tissue and specific fatty acids in the serum [18, 19]. In a cohort of patients with polycystic ovarial syndrome, we could identify a high rate of patients with NASH utilizing this assay [20]. Lately, the Feldstein group enhanced sensitivity and specificity of NASH detection by detection of soluble FAS and FAS ligand additional to M30 and established an apoptotic index in the noninvasive diagnosis of NASH [21]. 

As mentioned above, we found not only a correlation of M30 with hepatic steatosis but also a good correlation between the apoptosis rate and markers for fibrosis in our cohort, which is in line with observations from other groups [22]. Hepatocyte apoptosis is known to activate hepatic stellate cells via formation of apoptotic bodies, which are either directly stimulating hepatic stellate cells or are engulfed by Kupffer cells, which in turn activate stellate cells [23, 24]. Noninvasive diagnosis of fibrosis in NAFLD is complicated by the high rate of obese patients compared to hepatitis C infection. Transient elastography, a method that accurately predicts fibrosis stage in hepatitis C patients, faces limitations in NASH patients, as accuracy is dramatically reduced in obese individuals [25]. Thus, serum biomarkers might be a promising alternative in NAFLD patients. Hyaluronic acid has been implied as a good biomarker for fibrosis and was correlated with the apoptosis rate in our cohort [9]. However, several multipanel tests are among the most promising in noninvasive diagnosis of fibrosis. These tests consist of different serum parameters, some even include assessment of hyaluronic acid and are validated in cohorts of hepatitis C patients but might as well be useful in patients with NAFLD, although validation studies still need to prove accuracy in this cohort [26, 27].

## 5. Conclusion

Taken together, because of the nature of NAFLD and the increase in prevalence, there is an increasing demand for noninvasive markers of NAFLD and fibrosis. Since hepatocyte apoptosis is a key feature of NAFLD and also contributes to fibrogenesis, assessment of soluble markers of apoptosis appears to be a good alternative to liver biopsy in these patients. In fact, these methods might give better insight into the status and prognosis of liver disease than standard H&E assessment. However, especially fibrotic markers need further validation since the high rate of obesity might confound these tests.

## Figures and Tables

**Table 1:** Characterization of NASH and NAFL patients.

	NAFL (*n* = 71)	NASH (*n* = 56)	*P* value
Age (yr)	44.6 ± 1.3	40.7 ± 1.9	n.s.
Gender (w)	56 (79%)	44 (79%)	n.s.
BMI (kg/m^2^)	53.6 ± 0.9	51.2 ± 1.1	n.s.
Fasting-glucose (mg/dL)	106.8 ± 5.2	115.3 ± 6.6	n.s.
HbA1c (%)	5.8 ± 0.1	6.2 ± 0.2	n.s.
Total cholesterol (mg/dL)	191.3 ± 4.1	194.5 ± 5.7	n.s.
HDL cholesterol (mg/dL)	43.3 ± 1.2	46.1 ± 2.1	n.s.
LDL cholesterol (mg/dL)	130.7 ± 3.8	121.2 ± 5.4	n.s.
Triglycerides (mg/dL)	152.6 ± 21.0	157.3 ± 15.2	n.s.

Data are given as mean ± SEM or percentage affected. BMI: Body Mass Index.

## Abbreviations

NAFLD: Nonalcoholic fatty liver disease
NAS: Nonalcoholic fatty liver disease histological score
NASH: Nonalcoholic steatohepatitis.

## References

[1] Feldstein AE (2010). Novel insights into the pathophysiology of nonalcoholic fatty liver disease. *Seminars in Liver Disease*.

[2] Cheung O, Sanyal AJ (2008). Abnormalities of lipid metabolism in nonalcoholic fatty liver disease. *Seminars in Liver Disease*.

[3] Ertle J, Dechêne A, Sowa JP (2011). Non-alcoholic fatty liver disease progresses to hepatocellular carcinoma in the absence of apparent cirrhosis. *International Journal of Cancer*.

[4] Feldstein AE, Canbay A, Angulo P (2003). Hepatocyte apoptosis and fas expression are prominent features of human nonalcoholic steatohepatitis. *Gastroenterology*.

[5] Wieckowska A, Zein NN, Yerian LM, Lopez AR, McCullough AJ, Feldstein AE (2006). In vivo assessment of liver cell apoptosis as a novel biomarker of disease severity in nonalcoholic fatty liver disease. *Hepatology*.

[6] Yilmaz Y, Dolar E, Ulukaya E (2007). Soluble forms of extracellular cytokeratin 18 may differentiate simple steatosis from nonalcoholic steatohepatitis. *World Journal of Gastroenterology*.

[7] Feldstein AE, Wieckowska A, Lopez AR, Liu YC, Zein NN, McCullough AJ (2009). Cytokeratin-18 fragment levels as noninvasive biomarkers for nonalcoholic steatohepatitis: a multicenter validation study. *Hepatology*.

[8] McHutchison JG, Blatt LM, de Medina M (2000). Measurement of serum hyaluronic acid in patients with chronic hepatitis C and its relationship to liver histology. *Journal of Gastroenterology and Hepatology*.

[9] Nobili V, Alisi A, Torre G (2010). Hyaluronic acid predicts hepatic fibrosis in children with nonalcoholic fatty liver disease. *Translational Research*.

[10] Kleiner DE, Brunt EM, Van Natta M (2005). Design and validation of a histological scoring system for nonalcoholic fatty liver disease. *Hepatology*.

[11] NLM http://www.pubmed.gov.

[12] Canbay A, Friedman S, Gores GJ (2004). Apoptosis: the nexus of liver injury and fibrosis. *Hepatology*.

[13] Witek RP, Stone WC, Karaca FG (2009). Pan-caspase inhibitor VX-166 reduces fibrosis in an animal model of nonalcoholic steatohepatitis. *Hepatology*.

[14] Seeff LB, Everson GT, Morgan TR (2010). Complication rate of percutaneous liver biopsies among persons with advanced chronic liver disease in the HALT-C trial. *Clinical Gastroenterology and Hepatology*.

[15] Harwood J, Bishop P, Liu H, Nowicki M (2010). Safety of blind percutaneous liver biopsy in obese children a retrospective analysis. *Journal of Clinical Gastroenterology*.

[16] Ratziu V, Charlotte F, Heurtier A (2005). Sampling variability of liver biopsy in nonalcoholic fatty liver disease. *Gastroenterology*.

[17] Diab DL, Yerian L, Schauer P (2008). Cytokeratin 18 fragment levels as a noninvasive biomarker for nonalcoholic steatohepatitis in bariatric surgery patients. *Clinical Gastroenterology and Hepatology*.

[18] Bechmann LP, Gieseler RK, Sowa JP (2010). Apoptosis is associated with CD36/fatty acid translocase upregulation in non-alcoholic steatohepatitis. *Liver International*.

[19] Tabuchi M, Tomioka K, Kawakami T (2010). Serum cytokeratin 18 M30 antigen level and its correlation with nutritional parameters in middle-aged Japanese males with nonalcoholic fatty liver disease (NAFLD). *Journal of Nutritional Science and Vitaminology*.

[20] Tan S, Bechmann LP, Benson S (2010). Apoptotic markers indicate nonalcoholic steatohepatitis in polycystic ovary syndrome. *Journal of Clinical Endocrinology and Metabolism*.

[21] Tamimi TIAR, Elgouhari HM, Alkhouri N (2011). An apoptosis panel for nonalcoholic steatohepatitis diagnosis. *Journal of Hepatology*.

[22] Fitzpatrick E, Mitry RR, Quaglia A, Hussain MJ, DeBruyne R, Dhawan A (2010). Serum levels of CK18 M30 and leptin are useful predictors of steatohepatitis and fibrosis in paediatric NAFLD. *Journal of Pediatric Gastroenterology and Nutrition*.

[23] Gieseler RK, Marquitan G, Schlattjan M Hepatocyte apoptotic bodies encasing nonstructural HCV proteins amplify hepatic stellate cell activation: implications for chronic hepatitis C.

[24] Canbay A, Feldstein AE, Higuchi H (2003). Kupffer cell engulfment of apoptotic bodies stimulates death ligand and cytokine expression. *Hepatology*.

[25] Castéra L, Foucher J, Bernard PH (2010). Pitfalls of liver stiffness measurement: a 5-year prospective study of 13,369 examinations. *Hepatology*.

[26] Martínez SM, Crespo G, Navasa M, Forns X (2011). Noninvasive assessment of liver fibrosis. *Hepatology*.

[27] Ratziu V, Giral P, Munteanu M (2007). Screening for liver disease using non-invasive biomarkers (fibrotest, steatotest and nashtest) in patients with hyperlipidaemia. *Alimentary Pharmacology and Therapeutics*.

